# Vascular Dysfunction Predicts Future Deterioration of Left Ventricular Ejection Fraction in Patients with Heart Failure with Mildly Reduced Ejection Fraction

**DOI:** 10.3390/jcm10245980

**Published:** 2021-12-20

**Authors:** Shinji Kishimoto, Tatsuya Maruhashi, Masato Kajikawa, Takahiro Harada, Takayuki Yamaji, Yiming Han, Aya Mizobuchi, Yu Hashimoto, Kenichi Yoshimura, Yukiko Nakano, Kazuaki Chayama, Chikara Goto, Farina Mohamad Yusoff, Ayumu Nakashima, Yukihito Higashi

**Affiliations:** 1 Department of Cardiovascular Regeneration and Medicine, Research Institute for Radiation Biology and Medicine, Hiroshima University, Hiroshima 734-8551, Japan; shinji0922k@yahoo.co.jp (S.K.); 55maruchin@gmail.com (T.M.); tuplev144@gmail.com (Y.H.); mizobuchi@sanyo.ac.jp (A.M.); drfarinamyusoff@gmail.com (F.M.Y.); 2 Division of Regeneration and Medicine, Medical Center for Translational and Clinical Research, Hiroshima University Hospital, Hiroshima 734-8551, Japan; kajikawa5@hotmail.com (M.K.); keyoshim@hiroshima-u.ac.jp (K.Y.); 3 Department of Cardiovascular Medicine, Graduate School of Biomedical and Health Sciences, Hiroshima University, Hiroshima 734-8551, Japan; harataka0513@gmail.com (T.H.); ts5216yt@gmail.com (T.Y.); teriadeshio@yahoo.co.jp (Y.H.); nakanoy@hiroshima-u.ac.jp (Y.N.); 4 Department of Gastroenterology and Metabolism, Graduate School of Biomedical and Health Sciences, Hiroshima University, Hiroshima 734-8551, Japan; chayama@mba.ocn.ne.jp; 5 Department of Rehabilitation, Faculty of General Rehabilitation, Hiroshima International University, Hiroshima 739-2695, Japan; t-goto@hs.hirokoku-u.ac.jp; 6 Department of Stem Cell Biology and Medicine, Graduate School of Biomedical and Health Sciences, Hiroshima University, Hiroshima 734-8551, Japan; ayumu@hiroshima-u.ac.jp

**Keywords:** heart failure, vascular function, heart failure with mildly reduced ejection fraction, flow-mediated vasodilation, nitroglycerine-induced vasodilation

## Abstract

The purpose of this study was to evaluate whether heart failure with mildly reduced ejection fraction (HFmrEF) is associated with vascular dysfunction and whether vascular function predicts future deterioration of LVEF in patients with HFmrEF. We evaluated endothelial function assessed by flow-mediated vasodilation (FMD) and vascular smooth muscle function assessed by nitroglycerine-induced vasodilation (NID) in 69 patients with HFmrEF and 426 patients without HF and evaluated the future deterioration of LVEF, defined as a decrease in LVEF to <40%, in 39 patients with HFmrEF for up to 3 years. Both FMD and NID were significantly lower in patients with HFmrEF than in patients without HF. We categorized patients into two groups based on low tertiles of NID: a low group (NID of <7.0%) and an intermediate and high group (NID of ≥7.0%). There were significant differences between the Kaplan–Meier curves for the deterioration of LVEF in the two groups (*p* < 0.01). Multivariate Cox proportional hazard analysis revealed that NID of <7.0% was an independent predictor of future deterioration of LVEF in patients with HFmrEF. Both endothelial function and vascular smooth muscle function are impaired in patients with HFmrEF compared with those in patients without HF. In addition, low NID of <7.0% predicts future deterioration of LVEF.

## 1. Introduction

The mortality rate of patients with heart failure with mildly reduced ejection fraction (HFmrEF) is comparable to that of patients with HF with reduced ejection fraction (HFrEF) and that of HF patients with preserved ejection fraction (HFpEF) [1,2]. Previous studies have clearly shown that vascular dysfunction plays an important role in the pathogenesis and maintenance of HF including HFrEF and HFpEF [3,4,5,6,7]. Both patients with HFrEF and patients with HFpEF had vascular dysfunction compared with patients without HF [5,6]. Although it is thought that HFmrEF also has vascular dysfunction, it remains unclear whether HFmrEF is associated with vascular dysfunction. Changes in left ventricular EF (LVEF) in patients with HF are a common occurrence [8,9]. Deterioration of LVEF in patients with HFmrEF increases mortality and/or HF hospitalization [9]. It is clinically important to predict future deterioration of LVEF in patients with HFmrEF. Although there are few predictors for deterioration of LVEF in patients with HFmrEF [10,11,12], there is no established predictor for deterioration of LVEF.

Endothelial dysfunction is initially impaired in atherosclerosis and leads to the development and progression of atherosclerosis [13,14]. Endothelial function was measured by flow-mediated vasodilation (FMD) and vascular smooth muscle function was measured by nitroglycerine-induced vasodilation (NID) [15,16]. Both FMD and NID have been widely used due to being noninvasive. Growing evidence has shown that both FMD and NID can serve as independent predictors of cardiovascular events [17,18,19]. In addition, several studies showed that there were relationships of HF with vascular function assessed by FMD and NID and with vascular structure assessed by brachial intima-media thickness (IMT) and brachial–ankle pulse wave velocity (baPWV) [3,5,6,20].

The purpose of this study was to evaluate vascular function and vascular structure in patients with HFmrEF. In addition, we determined whether assessment of vascular function can be used for risk stratification regarding deterioration of LVEF in patients with HFmrEF.

## 2. Materials and Methods

### 2.1. Study Protocol 1

This study was a single-center, retrospective longitudinal cohort study. Between April 2010 and May 2020, a total of 240 patients with HF underwent FMD, NID, brachial IMT, and baPWV measurements and echocardiography, and 463 subjects without HF underwent FMD, NID, brachial IMT, and baPWV measurements and echocardiography. In total, 171 of the 240 patients who had HF, comprising 20 patients with severe renal dysfunction, 23 patients using nitrates, 44 patients with HFrEF, and 84 patients with HFpEF, were excluded. Finally, 69 patients with HFmrEF were enrolled in this study. We defined patients with no symptoms, no signs of HF, and either normal NT-proBNP or normal echocardiography on the basis of diagnostic criteria of the European Working Group for HF as patients without HF [21]. Furthermore, 37 of the 463 patients without HF, comprising 19 patients with severe renal dysfunction and 18 patients using nitrates, were excluded. Finally, 426 patients without HF were enrolled in this study.

Patients with HF were patients with symptoms or signs of HF and who were diagnosed with HF at Hiroshima University Hospital. HF was defined according to the diagnostic criteria of the European Society of Cardiology guidelines [21]. Patients with LVEF of ≥50%, LVEF of 40–49%, and LVEF of <40% were defined as patients with HFpEF, HfmrEF, and HFrEF, respectively.

Hypertension was defined as systolic blood pressure of more than 140 mmHg or diastolic blood pressure of more than 90 mmHg in a sitting position measured on at least three different occasions. Diabetes mellitus was defined according to the American Diabetes Association or a previous diagnosis of diabetes [22]. Dyslipidemia was defined according to the third report of the National Cholesterol Education Program [23].

We assessed vascular function using measurements of FMD and NID and vascular structure using measurements of brachial IMT and baPWV. Subjects fasted the previous night for at least 12 h and the study began at 8:30 a.m. The subjects were kept in a supine position in a quiet, dark, and air-conditioned room (constant temperature of 22–25 °C) throughout the study. A 23-gauge polyethylene catheter was inserted into the left deep antecubital vein to obtain blood samples. After thirty minutes of the subjects maintaining a supine position, we measured FMD, NID, brachial IMT, and baPWV. The observers were blind to the clinical status of the subjects and the purposes of the study [6].

All methods were carried out in accordance with relevant guidelines and regulations. The Ethics Review Board of Hiroshima University approved the study protocol (UMIN000003409). Written informed consent for participation in the study was obtained from all of the subjects.

### 2.2. Study Protocol 2

In this study, referred to as the follow-up study, we selected patients with HFmrEF for further study who had at least one additional transthoracic echocardiogram every year for up to three years after the baseline study. Finally, 39 patients with HFmrEF were enrolled in the follow-up study. The primary endpoint was deterioration of LVEF, defined as a decrease in LVEF to <40%.

### 2.3. Measurements of FMD and NID

Vascular response to reactive hyperemia in the brachial artery was used for assessment of endothelium-dependent FMD. A high-resolution linear artery transducer was coupled to computer-assisted analysis software (UNEXEF18G, UNEX Co, Nagoya, Japan) that used an automated edge detection system for measurement of brachial artery diameter [18]. The response to nitroglycerine was used for assessment of endothelium-independent vasodilation. NID was measured as described previously [18]. Additional details are available in Supplementary Materials.

### 2.4. Measurement of Brachial IMT

Before FMD measurement, baseline longitudinal ultrasonographic images of the brachial artery, obtained at the end of diastole from each of 10 cardiac cycles, were automatically stored on a hard disk for offline assessment of IMT with a linear, phased-array high-frequency (10 MHz) transducer using an UNEXEF18G ultrasound unit (UNEX Co) [24]. Additional details are available in Supplementary Materials.

### 2.5. Measurement of baPWV

Aortic compliance was assessed noninvasively on the basis of Doppler ultrasound measurements of PWV along the descending thoracoabdominal aorta, as previously reported and validated [25]. Briefly, baPWV, an index of arterial stiffness, was determined by two pressure sensors placed on the right ankle and left brachial arteries to record each pulse wave simultaneously (Form PWV/ABI, model BP-203RPE, Colin Co., Tokyo, Japan). The distance (D) between the two recording sensors was calculated automatically by inputting the value of individual height. The PWV value was calculated as PWV = D/t. PWV was measured for five consecutive pulses, and averages were used for analysis.

### 2.6. Echocardiography

Echocardiograms were obtained by using a Philips iE33 (Philips Co. Ltd., Bothell, WA, USA) with a 1.0 to 5.0 MHz transducer (S5-1). Routine two-dimensional imaging examinations were performed in parasternal long-axis and short-axis views and apical two-chamber and four-chamber views. Left atrial (LA) volume was measured by the biplane area–length formula and indexed for body surface area. LV mass was calculated according to the Penn Convention. LVEF was calculated by the modified Simpson formula.

### 2.7. Statistical Analysis

Results are presented as means ± SD for continuous variables and as percentages for categorical variables. Statistical significance was set at a level of *p* < 0.05. Categorical variables were compared by means of the χ^2^ test. Continuous variables were compared by ANOVA. One-to-one propensity-score matching analyses were used to create matched pairs to investigate the associations of HFmrEF with vascular function and vascular structure. The propensity score was calculated for each patient on the basis of logistic regression analysis of the probability of HFmrEF including age, sex, body mass index, systolic blood pressure, hypertension (yes/no), dyslipidemia (yes/no), diabetes mellitus (yes/no), and current smoking (yes/no). With these propensity scores, two well-matched groups based on clinical characteristics were created with a caliper width of 0.02 for comparison of vascular function. Time-to-event end point analyses were performed by using the Kaplan–Meier method. We categorized subjects into two groups according to the low tertiles of NID (<7.0%). The log-rank test was used to compare the groups. We evaluated the associations of deterioration of LVEF with NID after adjustment for age, sex, and cardiovascular risk factors by using Cox proportional hazard regression analysis. The number of variables that could enter the multivariate model was limited using the *p* < m/10 rule to prevent overfitting of the model. The data were processed using JMP Pro version 15 (SAS Institute, Cary, NC, USA).

## 3. Results

### 3.1. Study Protocol 1: Vascular Function and Vascular Structure in Patients with HFmrEF

**Figure 1:**
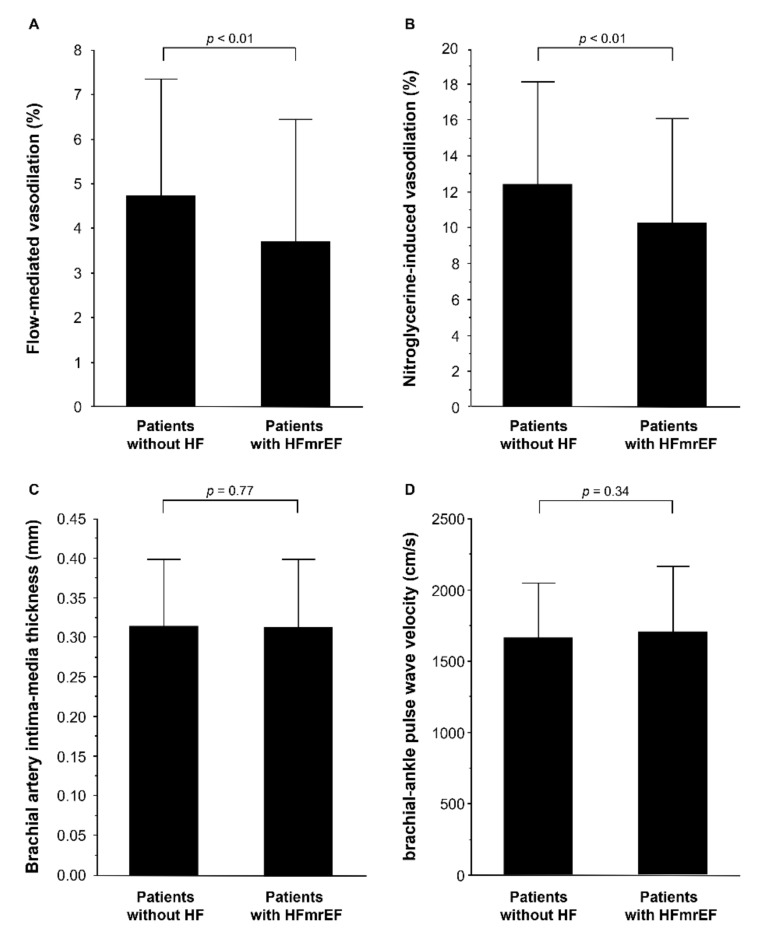
Bar graphs show flow-mediated vasodilation (**A**); nitroglycerine-induced vasodilation (**B**); brachial intima-media thickness (**C**), and brachial–ankle pulse wave velocity (**D**) in patients without heart failure (HF) and patients with heart failure with mildly reduced ejection fraction (HFmrEF).

The baseline clinical characteristics of the patients are summarized in Table 1. Of the 495 patients, 310 (62.6%) were men, 376 (76.1%) had hypertension, 315 (63.6%) had dyslipidemia, 135 (27.2%) had diabetes mellitus, 114 (23.1%) had previous coronary heart disease, and 76 (15.5%) were current smokers. Echocardiographic parameters of the 426 patients without HF and 69 patients with HFmrEF are summarized in Table 1. LV end-diastolic dimension index, LV end-systolic dimension index, LV mass index, and LA volume index were significantly higher in patients with HFmrEF than in patients without HF. EF was significantly lower in patients with HFmrEF than in patients without HF. FMD was significantly lower in patients with HFmrEF than in patients without HF (3.7 ± 2.7% versus 4.7 ± 2.5%, *p* < 0.01; Figure 1A). NID was significantly lower in patients with HFmrEF than in patients without HF (10.3 ± 5.9% versus 12.7 ± 5.7%, *p* < 0.01; Figure 1B). There was no significant difference in brachial IMT (0.32 ± 0.09 mm versus 0.32 ± 0.09 mm, *p* = 0.77; Figure 1C) and baPWV (1648 ± 372 cm/s versus 1705 ± 459 cm/s, *p* = 0.34; Figure 1D) between patients without HF and patients with HFmrEF.

**Figure 2:**
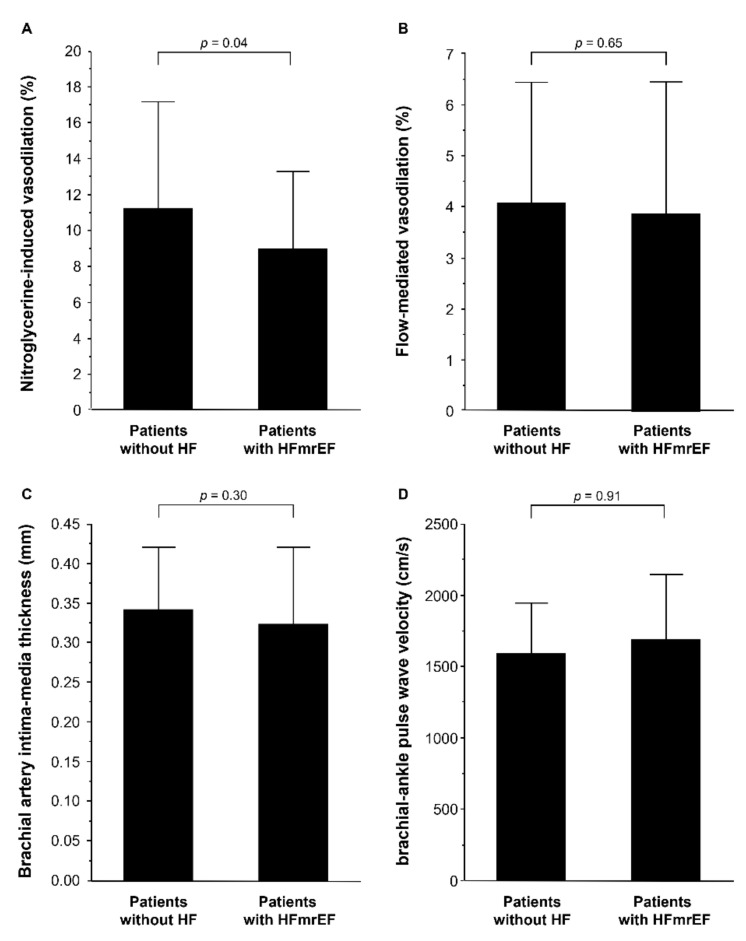
Bar graphs show nitroglycerine-induced vasodilation (**A**); flow-mediated vasodilation (**B**); brachial intima-media thickness (**C**), and brachial–ankle pulse wave velocity (**D**) in patients without heart failure (HF) and patients with heart failure with mildly reduced ejection fraction (HFmrEF) in propensity match population.

In addition, we evaluated vascular function in patients with HFmrEF and control subjects using the propensity score matching method to make matched pairs between patients without HF and patients with HFmrEF. The clinical characteristics of matched pairs of patients without HF and patients with HFmrEF are summarized in Table 2. NT-proBNP was significantly higher in patients with HFmrEF than in patients without HF. The percentage of patients using diuretics was significantly higher in patients with HFmrEF than in patients without HF. LV end-diastolic dimension index, LV end-systolic dimension index, and LV mass index were significantly higher in patients with HFmrEF than in patients without HF. LVEF was significantly lower in patients with HFmrEF than in patients without HF. NID was significantly lower in patients with HFmrEF than in patients without HF (8.9 ± 4.3% versus 11.2 ± 6.0%, *p* = 0.04; Figure 2A). There was no significant difference in FMD (4.0 ± 2.4% versus 3.8 ± 2.6%, *p* = 0.65; Figure 2B), brachial IMT (0.34 ± 0.08 mm versus 0.32 ± 0.10 mm, *p* = 0.30; Figure 2C), and baPWV (1594 ± 356 cm/s versus 1692 ± 458 cm/s, *p* = 0.91; Figure 2D) between patients without HF and patients with HFmrEF.

### 3.2. Study Protocol 2: Association of NID with Future Deterioration of LVEF in Patients with HFmrEF

The baseline clinical characteristics of the 39 patients with HFmrEF are summarized in Table 3. We categorized patients into two groups based on low tertiles of NID. The intermediate and high group had NID of ≥7.0% (12.4 ± 5.6%) and the low group had NID of <7.0% (4.0 ± 1.7%). There was no significant difference in other parameters between the two groups.

**Figure 3:**
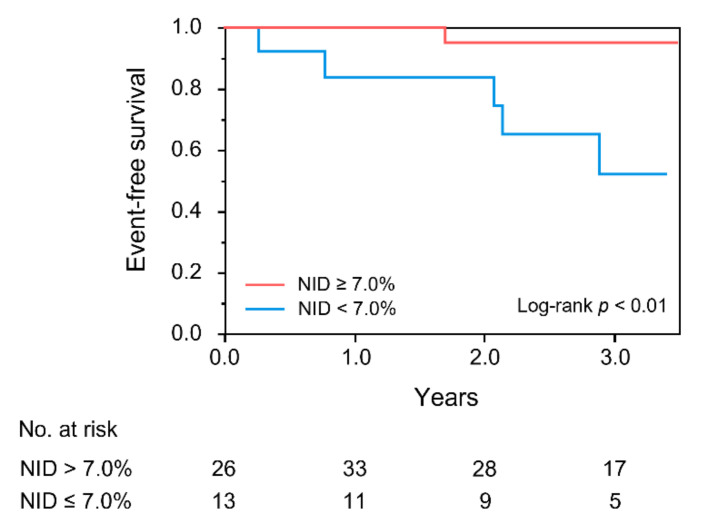
Kaplan–Meier curves of cumulative event-free survival of patients with heart failure with mildly reduced ejection fraction according to nitroglycerine-induced vasodilation (NID). The primary endpoint was deterioration of left ventricular ejection fraction (LVEF), defined as a decrease in LVEF to <40%.

During a median period of 2.6 years (interquartile range, 2.3–3.1 years) of follow-up, six patients had deteriorated LVEF. The Kaplan–Meier curves for the deterioration of LVEF between the two groups according to NID were significantly different (*p* < 0.01; Figure 3). Multivariate Cox proportional hazard analysis revealed that NID of <7.0% was an independent predictor of future deterioration of LVEF in patients with HFmrEF in Models 1 to 5 (Table 4).

## 4. Discussion

In the present study, we showed that both endothelial function assessed by FMD and vascular smooth muscle function assessed by NID were impaired in patients with HFmrEF compared with those in patients without HF, and we showed by using propensity score matching analysis that vascular smooth muscle function was impaired in patients with HFmrEF compared with that in control subjects. In addition, we demonstrated that NID of <7.0% was an independent predictor of future deterioration of LVEF in patients with HFmrEF. Vascular structure assessed by brachial IMT and baPWV was similar in patients with HFmrEF and patients without HF. These findings suggest that vascular function, but not vascular structure, is impaired in patients with HFmrEF and that measurements of NID might be useful for prediction of future deterioration of LVEF in patients with HFmrEF.

Previous studies have clearly shown that endothelial dysfunction plays an important role in the pathogenesis and maintenance of HF, especially in patients with HFrEF and patients with HFpEF [3,4,5,6]. In patients with HFrEF, endothelial dysfunction is induced by increases in oxidative stress and neurohumoral activity, alteration of shear stress, and a decrease in nitric oxide (NO) production [26,27]. In addition, endothelial dysfunction contributes to worsening HF via impaired myocardial perfusion and ventricular function, leading to a vicious circle between endothelial dysfunction and worsening HF in patients with HFrEF. Furthermore, in patients with HFpEF, several studies (including our study) have shown that endothelial function is impaired [6,28]. Endothelial dysfunction contributes to worsening HF via delayed myocardial relaxation and impairment of vascular compliance in patients with HFpEF. However, there is no information on vascular function in patients with HFmrEF. In the present study, we found that patients with HFmrEF had both endothelial dysfunction and vascular smooth muscle dysfunction. Some possible mechanisms by which vascular dysfunction might contribute to the pathogenesis and maintenance of HFmrEF are postulated. HFmrEF may also promote vascular dysfunction via increases in oxidative stress and inflammation. Superoxide suppresses not only NO production from endothelial cells but also intracellular signaling pathways in vascular smooth muscle cells via suppressing the activity of soluble guanylyl cyclase and cGMP-dependent kinase [29]. In addition, inflammation increases the connective tissue matrix in intima–media layers of vascular smooth muscle [30,31], leading to decreases in vascular relaxation responses to endogenous and exogenous NO.

Changes in LVEF in patients with HF are a common occurrence [8]. Some investigators showed that more than one-third of patients with HFmrEF had deterioration of LVEF [9,10]. Deterioration of LVEF in patients with HFmrEF is associated with mortality and/or HF hospitalization [9]. Therefore, it is clinically important to predict future deterioration of LVEF in patients with HFmrEF. However, it is difficult to predict future deterioration of LVEF in patients with HFmrEF [11,12]. Chang et al. showed that LV global longitudinal strain was associated with LVEF changes [11]. In contrast, measurement of LV global longitudinal strain failed to predict all-cause mortality and hospitalization for HF. Tsuji et al. showed that history of ischemic heart disease and LV dilatation were significantly associated with changes in LVEF in patients with HFmrEF [10]. These findings suggest that predictors of future deterioration of LVEF in patients with HFmrEF have not been established. It is well known that vascular dysfunction is closely correlated with HF [5,6,26]. In addition, assessments of vascular function have been shown to be predictors for cardiovascular events in patients with cardiovascular disease including patients with HF [32,33]. Previously, we confirmed that NID as well as FMD was independently associated with future cardiovascular events, including healthy subjects and patients with cardiovascular disease [34]. In the present study, low NID values were predictors of future deterioration of LVEF in patients with HFmrEF. Measurement of NID may be useful for prediction of mortality and/or HF hospitalization in patients with HFmrEF. Furthermore, in patients with HFmrEF, it is likely that there is a vicious circle between vascular dysfunction and the condition of HF. Therefore, restoration of vascular function by interventions including pharmacological treatment and lifestyle modifications may prevent worsening of HF.

There are some limitations in the present study. The number of patients with HFmrEF in the follow-up study was relatively small. However, we found that NID was a predictor of future deterioration of LVEF in patients with HFmrEF. The results of this study need to be confirmed in large clinical trials in patients with HFmrEF. In addition, we had no data on NID during follow-up periods. It is unclear whether there is a change in vascular function in patients with HFmrEF during the course of treatment for HF. Further studies are needed to confirm the effects of treatment for HF in patients with HFmrEF on vascular function.

## 5. Conclusions

In conclusion, vascular smooth muscle function is impaired in patients with HFmrEF compared with that in patients without HF. In addition, NID of <7.0% may be an independent predictor of future deterioration of LVEF in patients with HFmrEF. We should pay attention to patients with HFmrEF who have low NID during treatment for HF.

## Figures and Tables

**Table 1:** Clinical characteristics of the subjects correspond to Study Protocol 1.

Variables	Total (*n* = 495)	Patients without HF (*n* = 426)	Patients with HFmrEF (*n* = 69)	*p* Value
Age, year	61 ± 15	60 ± 15	66 ± 13	<0.01
Sex, men/women	310/185	257/169	53/16	<0.01
Body mass index, kg/m^2^	24.0 ± 4.4	24.2 ± 4.4	22.8 ± 4.2	0.01
Systolic blood pressure, mmHg	133 ± 20	134 ± 20	127 ± 21	<0.01
Diastolic blood pressure, mmHg	78 ± 12	79 ± 12	75 ± 14	0.01
Heart rate, bpm	71 ± 13	71 ± 13	73 ± 15	0.27
Total cholesterol, mmol/L	4.8 ± 1.0	4.9 ± 1.0	4.6 ± 0.9	0.03
Triglycerides, mmol/L	1.5 ± 1.0	1.5 ± 1.0	1.5 ± 1.1	0.59
HDL cholesterol, mmol/L	1.5 ± 0.5	1.6 ± 0.5	1.4 ± 0.4	<0.01
LDL cholesterol, mmol/L	2.8 ± 0.9	2.8 ± 0.9	2.7 ± 0.8	0.26
Glucose, mmol/L	6.2 ± 1.9	6.1 ± 1.6	6.7 ± 2.5	0.01
Hemoglobin A1c, %	5.7 ± 0.8	5.6 ± 0.8	6.1 ± 1.1	<0.01
Blood urea nitrogen, mmol/L	5.7 ± 1.9	5.4 ± 2.0	7.1 ± 2.5	<0.01
Creatinine, μmol/L	71.6 ± 20.3	69.8 ± 17.7	88.4 ± 28.3	<0.01
NT-proBNP, pg/mL	402 ± 873	199 ± 293	1373 ± 1704	<0.01
Etiology, n (%)				
Ischemic heart disease			30 (43.5)	
Dilated cardiomyopathy			2 (2.9)	
Hypertensive cardiomyopathy			8 (11.6)	
Valve disease			11 (15.9)	
Other			18 (26.1)	
Medical history, n (%)				
Hypertension	376 (76.1)	323 (75.8)	53 (76.8)	0.88
Dyslipidemia	315 (63.6)	263 (61.7)	52 (75.4)	0.03
Diabetes mellitus	135 (27.2)	112 (26.3)	23 (33.3)	0.23
Previous coronary heart disease	114 (23.1)	83 (19.5)	31 (44.9)	<0.01
Previous stroke	49 (10.0)	37 (8.8)	12 (17.4)	0.04
Current smoker, n (%)	76 (15.5)	64 (15.0)	12 (17.4)	0.64
Medication, n (%)				
Antiplatelets	143 (29.1)	111 (26.1)	32 (46.4)	<0.01
Calcium channel blockers	222 (45.2)	203 (47.7)	19 (27.5)	<0.01
ACEI or ARB	203 (41.3)	156 (36.7)	47 (68.1)	<0.01
β-blockers	121 (24.6)	81 (19.0)	40 (58.0)	<0.01
Diuretics	59 (12.0)	31 (7.3)	28 (40.6)	<0.01
Statins	199 (40.5)	160 (37.6)	39 (56.5)	<0.01
Nitrates	0 (0.0)	0 (0.0)	0 (0.0)	
Medically treated diabetes mellitus				
Any	87 (17.7)	77 (18.1)	10 (14.5)	0.44
Insulin dependent	22 (4.5)	15 (3.5)	7 (10.1)	0.03
Echocardiography				
LV ejection fraction, %	61 ± 7	63 ± 5	45 ± 3	<0.01
LV end-diastolic dimension index, mm/m^2^	30 ± 1	29 ± 4	32 ± 6	<0.01
LV end-systolic dimension index, mm/m^2^	20 ± 8	19 ± 3	25 ± 4	<0.01
LV mass index, g/m^2^	95 ± 30	90 ± 23	126 ± 47	<0.01
LA volume index, mL/m^2^	36 ± 13	35 ± 11	46 ± 19	<0.01

HF, heart failure; HFmrEF, heart failure with mildly reduced ejection fraction; HDL, high-density lipoprotein; LDL, low-density lipoprotein; NT-proBNP, N-terminal pro-brain natriuretic peptide; ACEI, angiotensin-converting enzyme inhibitor; ARB, angiotensin II receptor blocker; LV, left ventricular; LA, left atrial. Results are presented as means ± SD for continuous variables and percentages for categorical variables.

**Table 2:** Clinical characteristics of well-matched pairs of the subjects correspond to study protocol 1.

Variables	Patients without HF (*n* = 55)	Patients with HFmrEF (*n* = 55)	*p* Value
Age, year	67 ± 9	65 ± 14	0.51
Sex, men/women	43/12	42/13	0.82
Body mass index, kg/m^2^	23.3 ± 3.8	22.7 ± 4.3	0.44
Systolic blood pressure, mmHg	128 ± 18	129 ± 22	0.78
Diastolic blood pressure, mmHg	75 ± 11	76 ± 15	0.80
Heart rate, bpm	69 ± 11	73 ± 15	0.05
Total cholesterol, mmol/L	4.6 ± 0.8	4.6 ± 0.8	0.96
Triglycerides, mmol/L	1.5 ± 1.1	1.6 ± 1.2	0.68
HDL cholesterol, mmol/L	1.5 ± 0.4	1.4 ± 0.4	0.16
LDL cholesterol, mmol/L	2.6 ± 0.7	2.7 ± 0.8	0.23
Glucose, mmol/L	6.2 ± 1.4	6.9 ± 2.7	0.55
Hemoglobin A1c, %	5.8 ± 1.0	6.1 ± 1.3	0.23
Blood urea nitrogen, mmol/L	6.4 ± 2.4	7.1 ± 2.8	0.15
Creatinine, μmol/L	81.3 ± 27.4	86.6 ± 30.1	0.36
NT-proBNP, pg/mL	245 ± 399	1390 ± 1779	<0.01
Etiology, n (%)			
Ischemic heart disease		23 (41.8)	
Dilated cardiomyopathy		1 (1.8)	
Hypertensive cardiomyopathy		8 (14.6)	
Valve disease		10 (18.2)	
Other		13 (23.6)	
Medical history, n (%)			
Hypertension	46 (83.6)	44 (80.0)	0.62
Dyslipidemia	40 (72.7)	39 (70.9)	0.83
Diabetes mellitus	19 (34.6)	20 (36.3)	0.84
Previous coronary heart disease	20 (36.4)	23 (41.8)	0.56
Previous stroke	10 (18.2)	12 (21.8)	0.63
Current smoker, n (%)	9 (16.4)	11 (20.0)	0.62
Medication, n (%)			
Antiplatelets	19 (34.6)	23 (41.8)	0.43
Calcium channel blockers	24 (43.6)	15 (27.3)	0.07
ACEI or ARB	34 (61.8)	37 (67.3)	0.55
β-blockers	21 (38.2)	29 (52.7)	0.12
Diuretics	11 (20.0)	22 (40.0)	0.02
Statins	28 (50.9)	27 (49.1)	0.85
Nitrates	0 (0.0)	0 (0.0)	
Medically treated diabetes mellitus			
Any	15 (27.3)	9 (16.4)	0.16
Insulin dependent	3 (5.5)	7 (12.7)	0.18
Echocardiography			
LV ejection fraction, %	61 ± 6	45 ± 3	<0.01
LV end-diastolic dimension index, mm/m^2^	29 ± 4	32 ± 5	<0.01
LV end-systolic dimension index, mm/m^2^	19 ± 3	25 ± 5	<0.01
LV mass index, g/m^2^	97 ± 35	127 ± 48	<0.01
LA volume index, mL/m^2^	38 ± 16	48 ± 19	0.59

HF, heart failure; HfmrEF, heart failure with mildly reduced ejection fraction; HDL, high-density lipoprotein; LDL, low-density lipoprotein; NT-proBNP, N-terminal pro-brain natriuretic peptide; ACEI, angiotensin-converting enzyme inhibitor; ARB, angiotensin II receptor blocker; LV, left ventricular; LA, left atrial. Results are presented as means ± SD for continuous variables and percentages for categorical variables.

**Table 3:** Clinical characteristics of the patients with HFmrEF in follow-up study.

Variables	High and Intermediate NID (≥7.0%) (*n* = 26)	Low NID (<7.0%) (*n* = 13)	*p* Value
Age, year	62 ± 14	66 ± 16	0.45
Sex, men/women	23/3	8/5	0.06
Body mass index, kg/m^2^	23.0 ± 4.3	23.9 ± 4.1	0.55
Systolic blood pressure, mmHg	128 ± 20	133 ± 23	0.52
Diastolic blood pressure, mmHg	76 ± 12	74 ± 13	0.64
Heart rate, bpm	71 ± 11	78 ± 20	0.20
Total cholesterol, mmol/L	4.8 ± 0.8	4.3 ± 0.8	0.10
Triglycerides, mmol/L	1.8 ± 1.5	1.6 ± 0.9	0.79
HDL cholesterol, mmol/L	1.3 ± 0.3	1.5 ± 0.3	0.23
LDL cholesterol, mmol/L	2.9 ± 0.8	2.2 ± 0.5	0.03
Glucose, mmol/L	6.8 ± 2.1	8.0 ± 5.0	0.34
Hemoglobin A1c, %	6.3 ± 1.5	6.1 ± 0.3	0.67
Blood urea nitrogen, mmol/L	6.5 ± 2.4	7.6 ± 2.5	0.23
Creatinine, μmol/L	84.9 ± 28.3	100.8 ± 23.0	0.11
NT-proBNP, pg/mL	1114 ± 1741	1052 ± 634	0.91
Etiology, n (%)			
Ischemic heart disease	11 (42.3)	6 (46.2)	0.65
Dilated cardiomyopathy	2 (7.7)	0 (0.0)	0.20
Hypertensive cardiomyopathy	2 (7.7)	3 (23.1)	0.19
Valve disease	5 (19.2)	2 (15.4)	0.77
Other	6 (23.1)	2 (15.4)	0.57
Medical history, n (%)			
Hypertension	17 (65.4)	12 (92.3)	0.05
Dyslipidemia	21 (80.8)	10 (76.9)	0.78
Diabetes mellitus	10 (38.4)	4 (30.8)	0.63
Previous coronary heart disease	11 (42.3)	6 (46.2)	0.65
Previous stroke	2 (7.7)	3 (23.1)	0.19
Current smoker, n (%)	7 (26.9)	2 (15.4)	0.41
Medication, n (%)			
Antiplatelets	11 (42.3)	5 (38.5)	0.82
Calcium channel blockers	10 (38.5)	4 (30.8)	0.63
ACEI or ARB	17 (65.4)	9 (69.2)	0.81
β-blockers	14 (53.9)	8 (61.5)	0.65
Diuretics	9 (34.6)	4 (30.8)	0.81
Statins	14 (53.9)	7 (53.9)	1.00
Nitrates	0 (0.0)	0 (0.0)	
Medically treated diabetes mellitus			
Any	4 (15.4)	0 (0.0)	0.06
Insulin dependent	3 (11.5)	1 (7.7)	0.70
Echocardiography			
LV ejection fraction, %	44 ± 3	46 ± 3	0.16
LV end-diastolic dimension index, mm/m^2^	33 ± 5	31 ± 5	0.13
LV end-systolic dimension index, mm/m^2^	26 ± 4	24 ± 4	0.17
LV mass index, g/m^2^	128 ± 44	114 ± 48	0.38
LA volume index, mL/m^2^	42 ± 13	54 ± 25	0.07
FMD, %	3.7 ± 2.6	3.2 ± 2.6	0.58
NID, %	12.4 ± 5.6	4.0 ± 1.7	<0.01
Brachial IMT, mm	0.32 ± 0.10	0.32 ± 0.08	0.97
baPWV, cm/s	1655 ± 523	1916 ± 535	0.27

HFmrEF, heart failure with mildly reduced ejection fraction; NID, nitroglycerine-induced vasodilation; HDL, high-density lipoprotein; LDL, low-density lipoprotein; NT-proBNP, N-terminal pro-brain natriuretic peptide; ACEI, angiotensin-converting enzyme inhibitor; ARB, angiotensin II receptor blocker; LV, left ventricular; LA, left atrial; FMD, flow-mediated vasodilation; IMT, intima-media thickness; baPWV, brachial–ankle pulse wave velocity. Results are presented as means ± SD for continuous variables and percentages for categorical variables.

**Table 4:** Association between nitroglycerine-induced vasodilation and ejection fraction deteriorated during follow-up.

Variable	Unadjusted HR (95% CI) *p* Value	Model 1 HR (95% CI) *p* Value	Model 2 HR (95% CI) *p* Value	Model 3 HR (95% CI) *p* Value	Model 4 HR (95% CI) *p* Value	Model 5 HR (95% CI) *p* Value
NID ≥ 7.0%	1 (reference)	1 (reference)	1 (reference)	1 (reference)	1 (reference)	1 (reference)
NID < 7.0%	10.9 (1.3–93.7) 0.03	10.5 (1.2–92.1) 0.03	12.5 (1.0–152.5) 0.04	11.9 (1.3–106.3) 0.03	11.3 (1.2–102.6) 0.03	10.7 (1.2–93.8) 0.03

NID, nitroglycerine-induced vasodilation; HR, hazard ratio; CI, confidence interval. Hazard ratios are for <7.0% NID group, using the ≥7.0% NID group as the reference. Model 1: adjusted for age and sex. Model 2: adjusted for age, sex, and the presence of hypertension. Model 3: adjusted for age, sex, and the presence of dyslipidemia. Model 4: adjusted for age, sex, and the presence of diabetes mellitus. Model 5: adjusted for age, sex, and being a current smoker.

## Data Availability

The data presented in this study are available on request from the corresponding author. The data are not publicly available due to institutional policies requiring a data-sharing agreement.

## Supplementary Materials

The following are available online at https://www.mdpi.com/article/10.3390/jcm10245980/s1.
