# Supplementary material for: Vascular Dysfunction Predicts Future Deterioration of Left Ventricular Ejection Fraction in Patients with Heart Failure with Mildly Reduced Ejection Fraction
Source: J Clin Med. 2021 Dec 20;10(24):5980. doi: 10.3390/jcm10245980 (PMC8704434; doi:10.3390/jcm10245980)
Supplement: Supplementary file 1 [file jcm-10-05980-s001.zip › jcm-1448273-supplementary.pdf]

Online Supplement

Vascular Dysfunction Predicts Future Deterioration of Left Ventricular Ejection Fraction in Patients with Heart Failure with Mildly Reduced Ejection Fraction

Brief title: HFmrEF and vascular function

Shinji Kishimoto, MD, PhD;[1] Tatsuya Maruhashi, MD, PhD;[1] Masato Kajikawa, MD, PhD;[2] Takahiro Harada, MD;[3] Takayuki Yamaji, MD;[3] Yiming Han, MS;[1] Aya Mizobuchi, MS;[1] Yu Hashimoto, MD;[3] Kenichi Yoshimura, PhD;[2] Yukiko Nakano, MD, PhD;[3] Kazuaki Chayama, MD, PhD;[4] Chikara Goto, PhD;[5] Farina Mohamad Yusoff, MD;[1] Ayumu Nakashima, MD, PhD;[6] Yukihito Higashi, MD, PhD, FAHA[1,2]

[1]Department of Cardiovascular Regeneration and Medicine, Research Institute for Radiation Biology and Medicine, Hiroshima University, Hiroshima, Japan
[2]Division of Regeneration and Medicine, Medical Center for Translational and Clinical Research, Hiroshima University Hospital, Hiroshima, Japan
[3]Department of Cardiovascular Medicine, Graduate School of Biomedical and Health Sciences, Hiroshima University, Hiroshima, Japan
[4]Department of Gastroenterology and Metabolism, Graduate School of Biomedical and Health Sciences, Hiroshima University Hiroshima, Japan
[5]Dpartment of Rehabilitation, Faculty of General Rehabilitation, Hiroshima International University, Hiroshima, Japan
[6]Department of Stem Cell Biology and Medicine, Graduate School of Biomedical and Health Sciences, Hiroshima University Hiroshima, Japan

Address for correspondence: Yukihito Higashi, MD, PhD, FAHA
Department of Cardiovascular Regeneration and Medicine,
Research Institute for Radiation Biology and Medicine, Hiroshima University
1-2-3 Kasumi, Minami-ku, Hiroshima 734-8551, Japan
Phone: +81-82-257-5831 Fax: +81-82-257-5831
E-mail: yhigashi@hiroshima-u.ac.jp

Methods

Measurements of FMD and NID

Vascular response to reactive hyperemia in the brachial artery was used for assessment of endothelium-dependent FMD. A high-resolution linear artery transducer was coupled to computer-assisted analysis software (UNEXEF18G, UNEX Co, Nagoya, Japan) that used an automated edge detection system for measurement of brachial artery diameter.[1] A blood pressure cuff was placed around the forearm. The brachial artery was scanned longitudinally 5-10 cm above the elbow. When the clearest B-mode image of the anterior and posterior intimal interfaces between the lumen and vessel wall was obtained, the transducer was held at the same point throughout the scan by a special probe holder (UNEX Co) to ensure consistency of the image. Depth and gain setting were set to optimize the images of the arterial lumen wall interface. When the tracking gate was placed on the intima, the artery diameter was automatically tracked, and the waveform of diameter changes over the cardiac cycle was displayed in real time using the FMD mode of the tracking system. This allowed the ultrasound images to be optimized at the start of the scan and the transducer position to be adjusted immediately for optimal tracking performance throughout the scan. Pulsed Doppler flow was assessed at baseline and during peak hyperemic flow, which was confirmed to occur within 15 seconds after cuff deflation. Blood flow velocity was calculated from the color Doppler data and was displayed as a waveform in real time. The baseline longitudinal image of the artery was acquired for 30 seconds, and then the blood pressure cuff was inflated to 50 mm Hg above systolic pressure for 5 minutes. The longitudinal image of the artery was recorded continuously until 5 minutes after cuff deflation. Pulsed Doppler velocity signals were obtained for 20 seconds at baseline and for 10 seconds immediately after cuff deflation. Changes in brachial artery diameter were immediately expressed as percentage change relative to the vessel diameter before cuff inflation. FMD was automatically calculated as the percentage change in peak vessel diameter from the baseline value. Percentage of FMD [(Peak diameter - Baseline diameter)/Baseline diameter] was used for analysis. Blood flow volume was calculated by multiplying the Doppler flow velocity (corrected for the angle) by heart rate and vessel cross-sectional area (-r2). Reactive hyperemia was calculated as the maximum percentage increase in flow after cuff deflation compared with baseline flow.

The response to nitroglycerine was used for assessment of endothelium-independent vasodilation. NID was measured as described previously.[1] Briefly, after acquiring baseline rest images for 30 seconds, a sublingual tablet (75 µg nitroglycerine) was given, and images of the artery was recorded continuously until the dilation reached a plateau after administration of nitroglycerine. Subjects who had received nitrate treatment and subjects in whom the sublingually administered nitroglycerine tablet was not dissolved during the measurement were excluded from this study. NID was automatically calculated as a percent change in peak vessel diameter from the baseline value. Percentage of NID [(Peak diameter - Baseline diameter)/Baseline diameter] was used for analysis. Inter- and intra-coefficients of variation for the brachial artery diameter were 1.6% and 1.4%, respectively, in our laboratory.

Measurement of Brachial IMT

Before FMD measurement, baseline longitudinal ultrasonographic images of the brachial artery, obtained at the end of diastole from each of 10 cardiac cycles, were automatically stored on a hard disk for off-line assessment of IMT with a linear, phased-array high-frequency (10-MHz) transducer using an UNEXEF18G ultrasound unit (UNEX Co).[2] Measurement of IMT was automatically performed on A-mode images of the far wall of the brachial artery. The analysis system automatically chose the measurement point where an image of the posterior intimal interface was clearly obtained. If the measurement point was inappropriate, another clear image site could be manually selected for measurement. A total of 21 points over a 3-mm length of IMT in the 10-mm longitudinal image depicted in the analysis display were measured and the mean value per image was automatically calculated. IMT was measured at the same point in each image.

The average of mean values obtained from 10 cardiac cycles was defined as IMT of the brachial artery.

When measuring carotid IMT, we had an anatomical landmark, such as the carotid-artery bulb. Unfortunately, it is difficult to measure the same site of the brachial artery attributable to the lack of an anatomical landmark. Measurement of IMT in the brachial artery was performed at the proper site where the clearest B-mode image of the anterior and posterior intimal interfaces between the lumen and vessel wall was obtained at 5 to 10 cm above the elbow. However, there was little influence of intra- and interpatient variability in the measurement location of the brachial artery at 5 to 10 cm above the elbow in the present study, because the interface on the intima media of brachial artery is relatively smooth and intima-media thickening is not localized or plaques are not presented, resulting in diffuse intima-media thickening. The coefficients of variation of intra- and interobserver brachial IMT measurements were 3.1% and 4.0%, respectively.

References

1. Maruhashi T, Soga J, Fujimura N, Idei N, Mikami S, Iwamoto Y, Kajikawa M, Matsumoto T, Hidaka T, Kihara Y, Chayama K, Noma K, Nakashima A, Goto C, Higashi Y. Nitroglycerine-induced vasodilation for assessment of vascular function: A comparison with flow-mediated vasodilation. *Arteriosclerosis, thrombosis, and vascular biology*. 2013;33:1401-1408.
2. Iwamoto Y, Maruhashi T, Fujii Y, Idei N, Fujimura N, Mikami S, Kajikawa M, Matsumoto T, Kihara Y, Chayama K, Noma K, Nakashima A, Higashi Y. Intima-media thickness of brachial artery, vascular function, and cardiovascular risk factors. *Arteriosclerosis, thrombosis, and vascular biology*. 2012;32:2295-230.
